# Iterating the women in pain reduction and improved mood through empowerment intervention

**DOI:** 10.3389/fpain.2026.1859361

**Published:** 2026-07-06

**Authors:** Catherine Ann Clair, Natalie G. Regier, Sarah L. Szanton, Richard L. Skolasky, Janiece L. Taylor

**Affiliations:** 1 Johns Hopkins School of Nursing, Baltimore, MD, United States; 2 Johns Hopkins Medicine Division of Geriatric Medicine and Gerontology, Baltimore, MD, United States; 3 Department of Health Policy and Management, Johns Hopkins University Bloomberg School of Public Health, Baltimore, MD, United States; 4 Johns Hopkins Medicine Department of Orthopaedic Surgery, Baltimore, MD, United States

**Keywords:** depression, intervention, pain, pilot, repiloting, women

## Abstract

**Purpose:**

To explain key adaptations made to a depression and pain intervention that addresses inequities in pain outcomes among older women.

**Design:**

The DAPPER (Depression and Pain Perseverance through Empowered Recovery) pilot study and the Women in PRIME (Pain Reduction and Improved Mood through Empowerment) study were randomized controlled trials using waitlist control designs. DAPPER focused on pain and depressive symptoms among middle-aged and older African American women who were pre-frail or frail, while Women in PRIME addressed similar symptoms among middle-aged and older women of all races living with physical disabilities.

**Methods:**

Ten DAPPER and eight Women in PRIME participants were interviewed post-intervention completion. Qualitative data for both studies was analyzed using a deductive qualitative content analysis approach. Quantitative data included descriptive statistics.

**Results:**

Seven changes were made from the DAPPER Study to the Women in PRIME Study. These changes included 1) Re-naming the intervention; 2) Adding retention practices; 3) Expanding to include women of all races; 4) Switching criterion of (pre)frailty to physical disability; 5) Adding group Acceptance & Commitment Therapy (ACT) sessions; 6) Reducing individual nurse visits from 8 to 5 visits; and 7) Switching from saliva to sweat patch for biomarker measurement.

**Conclusions:**

The changes made from DAPPER to Women in PRIME resulted in greater retention, participation in the intervention, and participation in the data collection timepoints. A critical component of equity-focused intervention iteration is ensuring that participant input and guidance directly shape the modifications and adaptations that advance the intervention toward larger scale trials.

## 1 Introduction

### 1.1 Background on pain and depression

The co-occurrence of chronic pain and depression is a disparate experience that exists specifically among older women, largely due to factors such as age, sex, a high prevalence of comorbid conditions, and overlapping pathophysiological mechanisms (1, 2). Beyond biological links, they also exhibit similar clinical manifestations and often respond to comparable treatment approaches (2). When either or both conditions are left untreated in older women, the result can be a significantly diminished quality of life. Social determinants of health such as low income, limited access to mental health services, and social isolation further compound these inequities in pain faced by older women with comorbid conditions, particularly those from marginalized communities (3). Therefore, it is essential that both chronic pain and depression are thoroughly assessed and effectively managed in this population.

Given the multifaceted nature of pain and depression and the unique needs of older women with comorbid conditions, it is essential to develop health equity-focused interventions that are informed by participant input and tailored to their lived experiences. Incorporating feedback from the intended population not only enhances the contextual relevance of the intervention but also improves its acceptability and long-term sustainability (4). Interventions focused on equity in pain outcomes are more successful when they are informed by people with lived experience of pain and aligned with the communities they are meant to serve (5). Human-centered design methodologies can be used to design and refine interventions alongside participants rather than for participants. This type of design aids researchers in better understanding participants' needs and experiences and recognizing the barriers and facilitators from their perspective (6, 7). Additionally, older women with chronic, disabling conditions are often not included in larger-scale trials that address these conditions due to issues with access (e.g., transportation, physical accessibility) and/or inclusion criteria (e.g., upper age limits), which limits their inclusion in the broader existing literature (8). Prior to the original pilot of the Depression and Pain Perseverance through Empowered Recovery (DAPPER) Study, there were no studies that specifically addressed comorbid chronic pain and depression in older women.

### 1.2 Original pilot, continued iteration & objectives

The DAPPER Study piloted a behavioral intervention to address symptoms of pain and low mood among middle-aged and older African American women. Based on limitations in previous published literature, the inclusion criterion for age had no upper limit, and we enhanced access by offering intervention visits in-home or virtually. To develop a health equity-focused intervention, we used human-centered design techniques to elicit feedback from conception to completion of the intervention from women with lived experiences of pain and depressive symptoms. From this feedback, we repiloted and renamed the intervention to “Women in PRIME (Pain Reduction and Improved Mood through Empowerment).” In an effort to develop an effective sustainable intervention for pain and low mood, we conducted this second pilot (“repiloting”). Repiloting is a critical component of intervention development to ensure investments in a larger-scale trial are based on strong evidence from adaptations of the original pilot (9). The second pilot, the Women in PRIME Study, had several major changes to study process, procedure, and intervention content compared to its predecessor. While iterative testing is common for pilot studies and their subsequent scaling, it is rare for trials to report the changes made and their success. The purpose of this paper is to explain key adaptations made to the DAPPER Study for the Women in PRIME Study and provide relevant findings on the relative impact of these changes.

## 2 Methods

### 2.1 Study design and intervention

#### 2.1.1 DAPPER

The Depression and Pain Perseverance through Empowered Recovery (DAPPER) study was a randomized controlled trial using a waitlist control design for middle-aged and older African American women who were frail or pre-frail. The intervention consisted of eight in-person or virtual 1-hour visits with a nurse. The nurse worked with participants on their goals surrounding pain and depression using non-pharmacological, tailored strategies. The DAPPER Study protocol, including both intervention content and data collection measures, is published elsewhere (10). The DAPPER study protocol and procedures were approved by the [Johns Hopkins Medicine] Institutional Review Board (IRB00226182).

#### 2.1.2 Women in PRIME

The Women in Pain Reduction and Improved Mood through Empowerment (PRIME) Study was a randomized controlled trial using a waitlist control design. The intervention used non-pharmacological, tailored strategies to target pain and depressive symptoms among middle-aged and older women living with physical-function limitations. The inclusion criterion of functional limitations was assessed at screening (“Do you need help or personal assistance completing any of the following or are you unable to do any of the following: Bathe or shower, get dressed or undressed, use the restroom in your home or in a public restroom, transfer from sitting to standing or standing to sitting, getting in or out of the bed, climbing up a flight of stairs in your home or outside of your home, such as the mall, church, or other place?”). Participants had to have difficulty or needed to be unable to perform the task in at least one of the areas.

The intervention consisted of five in-person or virtual 1-hour visits with a nurse and six group Acceptance & Commitment Therapy (ACT) sessions led by a clinical psychologist. The Women in PRIME Study protocol, including both intervention content and data collection measures, is published elsewhere (11). The Women in PRIME study protocol and procedures were approved by the [Johns Hopkins Medicine] Institutional Review Board (IRB00341962).

### 2.2 Qualitative data collection

#### 2.2.1 DAPPER

After completing the DAPPER Study, a select subsample of participants (*n* = 10) completed semi-structured qualitative interviews to assess acceptability and feasibility of the intervention. All interviews were conducted by the first author, who has had extensive training in qualitative interviewing. Following the participant's consent to the recording, they were asked questions from a semi-structured interview guide. Initial questions were broad (“Tell me about your experience in the DAPPER program,” “What was your impression of the DAPPER program?”) to set the tone and offer a neutral stance to open the conversation (12, 13). To learn more about their experience, participants were then asked about positives (“What was most valuable or useful about the DAPPER program for you?”), and challenges (“What was most challenging about participating in the DAPPER program?”).

Additionally, they were asked about if and how the intervention had helped with their pain and mood. To elicit suggested changes, participants were asked to comment on 1) the number of nurse visits, 2) the length of the individual visit, 3) the preferred qualities for a nurse interventionist and study team members, and 4) their choice of visit modality. The study team also presented several proposed changes to the program, including adding group sessions and changing the program acronym, for the women to provide feedback.

While the DAPPER Study ran from November 2019—December 2023, semi-structured interviews occurred from January 2021 to May 2023. All the participants selected for interviews agreed to participate, and on average, interviews were 20 min (Range: 5–30 min). Following the interview, participants received a $10 gift card as a token of appreciation for their time and effort.

#### 2.2.2 Women in PRIME

After completing the Women in PRIME Study, all retained participants (*n* = 8) completed semi-structured qualitative interviews to assess acceptability and feasibility of the intervention. Again, all interviews were conducted by the first author. Similar to that of the DAPPER interview guide, initial questions were broad (“Tell me about your experience in the Women in PRIME program”) and then narrowed to assess the intervention's impact on their pain and mood symptoms, their feedback on intervention elements, and their response to various study elements, such as data collection, the study team, and retention efforts. Interviews occurred from May 2024 to January 2025 and on average were 40 min (Range: 30–60 min). Following the interview, participants received a $10 gift card as a token of appreciation for their time and effort.

### 2.3 Data analysis

#### 2.3.1 Qualitative

Interviews for both studies were audio-recorded, transcribed verbatim, and analyzed using a deductive qualitative content analysis approach (14, 15). Specifically, for each iteration, transcripts were examined by [CAC] to identify responses to specific guiding questions related to intervention acceptability, participant experience, and recommended changes. Responses were collated by question, and salient quotes were selected to illustrate key themes. Following this process, [CAC] and [JLT] met to review and discuss the findings and further investigate (dis)confirming experiences (16). No qualitative data management and organizational software was used; analysis was conducted manually using Microsoft Word to facilitate close reading and thematic extraction.

#### 2.3.2 Quantitative

For demographic characteristics, we ran descriptives statistics for the combined cohort and by study. Continuous variables are presented as mean and standard deviation, and categorical variables are presented as count (n) and percentages (%). Appropriate statistical tests (e.g., Mann–Whitney, Fisher's exact) were used to compare the two study samples. Significance level was set to *p* < 0.05.

For the objectives of this analysis, we ran descriptive statistics of study measures, including retention, intervention participation, and data collection participation. We present count (n) and percentages (%).

## 3 Results

Across the two studies, we enrolled 43 participants (Table 1). There were no significant differences between the study populations, excluding race (which served as an inclusion criterion for the DAPPER Study).

**Table 1:** Baseline characteristics of combined cohort and by study.

Characteristic	Combined (*N* = 43)	DAPPER Study (*n* = 34)	Women in PRIME Study (*n* = 9)	*p* value	Test
Age (Mean, SD)	64.4 (9.4)	64.8 (10.5)	62.8 (2.8)	0.72	Mann–Whitney
Race (*N*, %)				<0.001	Fisher's exact
Black or African American	36 (84)	34 (100)	2 (22)		
White	6 (14)	-	6 (67)		
Native American/Alaskan Native	1 (2)	-	1 (11)		
Average daily pain score at baseline (Mean, SD)	7.0 (1.9)	7.0 (1.9)	7.1 (2.3)	0.95	Mann–Whitney
PHQ-9 score at baseline (Mean, SD)	11.1 (4.5)	11.5 (4.4)	9.7 (4.6)	0.19	Mann–Whitney

Seven significant changes were made from the DAPPER Study to the Women in PRIME Study. These changes were grouped under four main domains: overall study elements, inclusion criteria, intervention components, and data collection. Each change is listed below with its corresponding justification, result, and lessons learned.

### 3.1 Overall study elements

#### 3.1.1 Updated intervention name: what's in a name?

- Justification: When asked about changing the name of the intervention, one DAPPER participant (Participant #D1) shared, “When we think of the word DAPPER– Dapper Dan. You think of a dashing man, somebody moving fast, doing things.” This feedback resulted in changing the intervention name from “Depression and Pain Perseverance through Empowered Recovery” to “Women in Pain Reduction and Improved Mood through Empowerment (PRIME)” (11). Another participant from DAPPER (Participant #D2) described the proposed new acronym as “specifically for women, so that's always good.”
- Results: The participants in the Women in PRIME Study were not explicitly asked to comment on the intervention name. However, one participant noted that when she tried to search for the study, other studies with similar acronyms (e.g., “PRIME”) were the first to appear.
- Lessons Learned: A name matters. Choosing an intervention title that participants could recognize, remember, and embrace increased their sense of ownership and identification with the program. Renaming the study, “Women in PRIME” resonated with participants and helped foster trust and inclusion. This is particularly important for groups, like middle-aged and older women, who have historically been underrepresented in clinical research (17).

#### 3.1.2 Added retention practices: meaningful communication

- Justification: During the DAPPER Study, monthly phone calls were only conducted from baseline (T0) to 12 weeks (T1) for the waitlist control group (10, 18). Overall, of the 34 participants consented in DAPPER, 25 (73.5%) completed the study. We added additional retention practices to the Women in PRIME study (11). This included a monthly newsletter—mailed or emailed depending on participant preference—and monthly phone calls during the “waiting” period for each group. For the waitlist control group, these calls occurred from baseline to 12 weeks. For the immediate intervention group, the calls occurred from post-intervention (12 weeks) to 24 weeks.
- Results: Of the nine participants enrolled in the Women in PRIME Study, eight (88.9%) completed the study. All eight participants completed the three phone calls. One participant in the immediate intervention group was lost to follow-up during their intervention period. When asked about the newsletter, participants responded affirmatively to receiving the newsletter; one participant noted that was “nice to get.” However, there were mixed responses to the content. Some participants described the content as “informative, “helpful,” and “interesting.” One participant (Participant #W1) stated: “I look forward to them. Every time I get them, I put them on the refrigerator..the newsletter is very, very nice. It's well done, simple, and positive.” In contrast, other participants felt that the newsletter lacked substance. One participant (Participant #W2) felt that “it just seemed like it was kind of fluff.
- Lessons Learned: We learned to prioritize the content participants found most valuable. Soliciting their input revealed what information and supports made them feel seen and included—not only during active intervention sessions but throughout the entire study experience. Incorporating these preferences improved relevance, engagement, and participants' sense of respect and inclusion.

### 3.2 Inclusion criteria

#### 3.2.1 Expanded to include women of all races: sense of belonging

- Justification: The inclusion criteria for the DAPPER study included identifying as an African American woman (10). We developed this intervention with a population experiencing the most need and inequities in pain outcomes. However, limiting to a single racial group reduces the scalability and reach of the intervention. Pain is a very common condition that impacts older adults of all races/ethnicities. One DAPPER participant (Participant #D3) shared that “she was wondering why a particular [racial] group” was the only one included in the intervention.
- Results: In the Women in PRIME Study, participants were asked about the “differences” they noted about the women in their group Acceptance & Commitment Therapy (ACT) sessions. None of the participants mentioned race; instead, participants noted differences in pain intensity and duration, life experience, and personality.
- Lessons Learned: Participants raised questions about race eligibility early in the DAPPER Study, asking why the original pilot included only African American women. We learned it was important to explain our rationale; centering a group disproportionately affected by pain and low mood and underrepresented in research is key to build transparency and trust. Equally important was communicating that the repilot expanded eligibility to include all races, and why that change was made. Clear, respectful explanations about inclusion criteria supported participant understanding and helped mitigate concerns about exclusion.

#### 3.2.2 Switched criterion of (pre)frailty to impaired function: shift from a disease to participation focus

- Justification: Many of the participants from the DAPPER Study described decreasing pain interference as a key marker of their progress. For example, one DAPPER participant (Participant #D2) shared, “I don't have to wait for somebody to come to get my things out of the washing machine so I can relocate them to the dryer…it's allowed me, given me some, of course, what I wanted to be able to do things on my own.” The accomplishments of the participants were primarily focused on functional ability, which suggested that physical ability may be more central to their experiences than frailty.
- Results: Of the 62 individuals screened for the Women in PRIME, 14 (22.6%) were excluded due to a lack of physical disability.
- Lessons Learned: Participant feedback showed that the intervention was affecting physical function and social participation, and women were setting goals in these areas. In response, we changed the inclusion criteria from (pre)-frailty to impaired physical function, so the study better reflected who may benefit from the intervention. Including women with impaired function was important both because pain is a leading cause of disability (19, 20) and because it ensured the sample matched the intervention's real-world impact and priorities. We also included measures that reflected physical disability and social participation.

### 3.3 Intervention components

#### 3.3.1 Added group acceptance & commitment therapy (ACT) sessions: building community

- Justification: We added six ACT group sessions to the intervention, based on the success of ACT in other behavioral interventions focused on pain (21, 22). DAPPER participants responded positively to the proposed addition of group sessions. One DAPPER participant (Participant #D4) described how “people learn more when it's more than a few, because what one person might not know someone else could have experienced and can enlighten you.” Another participant (Participant #D5) responded that she liked the addition because “it gives you a chance to meet, to see your friends and the persons you interact with.”
- Results: On average, Women in PRIME participants attended 4.3 sessions (range: 1–6). Four participants (44.4%) attended all six sessions. On average, participants completed 1.2 homework assignments (range: 0–3). Four participants did not complete any homework assignments.
- Lessons Learned: Participants in the DAPPER pilot expressed interest in a group component, so we added a group-based Acceptance and Commitment Therapy sessions in the Women in PRIME repilot. Uptake of the group sessions was mixed. This taught us to balance participant preferences with practical considerations: Group formats can enhance social support and relevance but require additional coordination, flexible scheduling, and strategies to maximize attendance.

#### 3.3.2 Reduced individual nurse visits from 8 to 5 visits: participant-centered decision-making

- Justification: With the addition of the six ACT sessions, the number of nurse visits was reduced from eight to five (10, 11). This decision aimed to reduce participant burden given the addition of the group sessions.
- Results: In the DAPPER Study, 26 (92.9%) participants who started the intervention completed all 8 visits. In the Women in PRIME Study, eight (88.9%) participants completed all 5 visits.
- Lessons Learned: We identified that the eight visits were more feasible from a study-coordination perspective than the five nurse visits coupled with the six group sessions. Participants were able to build a rapport with the nurse in both the pilot and repilot. We noted in the repilot that a few participants expressed the desire for more nurse visits.

### 3.4 Data collection

#### 3.4.1 Switched from saliva to sweat patch for biomarker measurement: equitable exploration of new methods

- Justification: The DAPPER Study aimed to demonstrate feasibility of saliva data collection (10). The DAPPER saliva data collection was feasible with 29 participants (85.3%) submitting at least one saliva sample and 23 participants (67.6%) completing saliva samples at both baseline and 12 weeks. Overall, DAPPER participants reported no feasibility issues with the saliva data collection. Some participants expressed confusion regarding the purpose of the saliva collection and its use in the study. The Women in PRIME Study aimed to demonstrate feasibility of sweat patch data collection and compare its efficiency to that of the saliva. To address the issue of confusion, we provided additional training to data collectors that included several stop-points to elicit participant questions and ensure their understanding of the biomarker measurement process.
- Results: Of the nine participants, eight were safely able to wear the sweat patch. Of the eight participants, seven (87.5%) completed both sweat patch data collections. When asked about the sweat patch data collection, participants used words like, “fine,” “appropriate,” and “wasn't hard.” One participant (Participant #W3) shared that she thought additional sweat patch data would be helpful to the study: “If you had more data points with the sweat patch, you could really see [change] throughout the program.” None of the WPRIME participants expressed confusion regarding the purpose of the sweat patch and its inclusion as a measure of study outcomes.
- Lessons Learned: We were able to utilize a new method of collecting biomarkers in this population, identifying that both saliva and sweat patches were feasible and acceptable for the women across the two studies. Additionally, regardless of the methods chosen, we found that communication about the next steps with this data was crucial.

## 4 Discussion

Across these studies, we sought to address pain and depressive symptoms among two populations of women who have historically experienced disparities in pain and mental health outcomes. This analysis illustrates the iterative process from an initial pilot intervention to a subsequent repilot (i.e., DAPPER to Women in PRIME). Across this progression, seven significant changes were implemented in the Women in PRIME study relative to its predecessor, spanning overall study elements, inclusion criteria, intervention components, and data collection procedures.

Several changes proved advantageous, in which the change positively impacted the Women in PRIME study metrics. First, the additional retention practices implemented in the Women in PRIME Study resulted in a 15.5 percentage point-difference in retained participants between Women in PRIME and DAPPER. Existing literature has pointed to reminders—defined as reminders about appointments and study participation—as a successful retention strategy (23). Utilizing passive and active reminders may have contributed to the success in retention rate. The monthly newsletter represented a passive reminder of study participation; every participant received the same newsletter. Communicating about the process of a clinical trial is often a missed step that can effectively help with recruitment and retention of African American women (19) and other underrepresented groups in clinical trials. In contrast, the monthly calls were a more active reminder of their study participation during wait periods. Second, there was a 19.9 percentage-point difference in completing the biomarker data collection method, comparing Women in PRIME to DAPPER. Saliva collection, which was used in the DAPPER Study, has been documented as a feasible and acceptable method of biomarker data collection (24, 25). For example, Emami and colleagues found that in-home saliva collection was feasible for individuals living with dementia and their caregivers (24). However, there is a dearth of literature assessing the acceptability and feasibility of sweat patch data collection, particularly among community-dwelling older adults. We demonstrated sweat patch feasibility and Women in PRIME participants' description of the process indicate efficiency. Future research should aim to assess acceptability and feasibility of sweat patch data collection as the primary objective.

Our decision to change inclusion criteria from pre-frailty/frailty to impaired physical function was not only associated with the goals of the women but also furthered our understanding of the outcomes associated with the intervention. Frailty is conceptualized as a syndrome involving multiple systems that is associated with increased physical decline in aging (26, 27). In contrast, functional limitations are present when an individual experiences difficulty completing physical, cognitive, or emotional tasks. Finally, disability is when a person can no longer fill social roles because of the difficulties associated with fulfilling the above mentioned tasks (27, 28). We identified that many of the goals set in the DAPPER study were associated with achieving tasks and improving social participation, which are more aligned with functional limitations and disability. Thus, we altered the inclusion criteria for the Women in PRIME repilot to include women experiencing functional limitations and/or disabilities. Existing literature highlights that pain and depression are directly related to function and disabilities (29–31). We also saw little differences in baseline pain and depressive symptoms reported by the women in both pilots, suggesting that the focus on function may be a more universal experience. This intervention may have more of an immediate and sustainable impact on regaining function and social participation than frailty.

The inclusion of added group ACT sessions was well received; on average, participants attended over half of the sessions offered with almost half attending all six sessions. This aligns with existing literature as various group-based virtual programming for older adults have demonstrated acceptability, feasibility, and effectiveness (32, 33). Specifically in the Women in PRIME Study, the virtual group sessions were centered on ACT, which has been proven feasible among older adults (21, 34). Theoretically, the impact of the ACT component of the Women in PRIME repilot is interwoven with the shift from an inclusion criterion of pre(frailty) to one of physical function. The new focus on social participation as part of disability, function, and goal setting was likely further promoted by the community building in the ACT sessions. This conceptual alignment may have strengthened the delivery of the repilot, creating a synergy between intervention design, inclusion criteria, and study outcomes. Still, there were several components of the ACT sessions that would benefit from continued iterations, such as the low completion rate for the weekly homework. Additional research may consider if homework is a necessary component and if yes, how to enhance participation and improve completion. While homework offers opportunity for participants to hone the skills learned, the value of the ACT sessions may lie in the community-building element.

Overall, repiloting is critical in the improvement of interventions from pilot to larger-scale trial. Beets and colleagues describe one of the common missteps in scaling interventions is neglecting to repilot (9). Repiloting is “engaging in more than one round of preliminary testing to enhance intervention effects or address identified obstacles” (35). This process offers the opportunity to provide stronger evidence that adaptations from the original pilot should be invested in within a larger-scale trial. While it is known that pilot studies provide necessary information for a larger-scale trial (36), repiloting is deeply valuable but less common. In the study by von Klinggraeff and colleagues (2022), of the 24 investigators interviewed, only five described repiloting their interventions (35).

Despite the lack of repiloting, intervention adaptation is a common phenomenon in implementation science. As interventions transition from pilot studies to larger clinical trials, adaptations to service setting, target audience, mode of delivery, and cultural contextual factors can be made (37). Chambers and Norton (2016) describe how simultaneously changing multiple factors from an original pilot without repiloting can result in discordance and lessen the contribution of the intervention to science when it is scaled up (37). To date, many scientists have focused on cultural adaptations, ensuring that an intervention designed for one group resonates and has similar impact with another (38, 39). Adaptations to age, race, ethnicity, culture, and language must be thoroughly considered and tested. However, it is also critical to test accessibility, dosage, intensity of the intervention, staffing, and other study-related elements (37). It is clear that a common misstep in intervention development is neglecting to repilot the intervention (9). By iterating (or “repiloting”) the DAPPER Study with Women in PRIME, we aim to bring stronger evidence of study process and procedures to a larger-scale trial. This is particularly essential when scaling interventions and programs aimed to meet and support populations affected by disparities in pain and depression management.

In pain research, a substantial body of literature documents persistent disparities in pain management outcomes, as well as related mental health outcomes, among marginalized populations (40–42). Advancing an equity-oriented approach to pain and mental health outcomes requires a sustained commitment to developing and rigorously testing interventions that are not only methodologically sound but also grounded in the lived experiences, expertise, and priorities of the populations most affected by these disparities. While clinicians are often guided by the principle that “pain is what the patient says it is,” this perspective is frequently underutilized in the design of pain management strategies. Incorporating patient experiences, perceptions, and responses alongside biological mechanisms is essential for the development of equitable pain research and clinical interventions (43). Moreover, interventions that simultaneously address pain and co-occurring chronic conditions, such as depressive symptoms, may offer greater sustainability and advance equity more effectively than approaches that focus on pain alone, particularly among populations experiencing a disproportionate burden of pain-related disparities (43).

### 4.1 Strengths & limitations

This analysis has several strengths. Reporting on the iterative nature of pilots is rare and presents an opportunity to better understand changes made in the progress to a larger trial. Secondly, we present both qualitative and quantitative results for the outcome of each change, offering greater insight into its success. However, given the small sample size of both studies, it is important to note that our findings are indicators of feasibility and acceptability rather than of intervention impact. Finally, the most significant limitation is the nature of the qualitative data, as this was a secondary qualitative analysis. The Women in PRIME interviews were not directly focused on all changes made from the DAPPER Study. While some elements were included in the interview guide, this was not the interview's primary objective and limits our understanding of several noted changes.

## 5 Conclusion

A critical component of equity-focused intervention iteration is ensuring that participant input and guidance directly shape the modifications and adaptations that will advance the intervention toward larger scale trials. Iterating pilot interventions is an important step to pursuing a larger intervention trial. From the DAPPER Study (original pilot) to its successor, the Women in PRIME Study (repilot), there were several significant changes made. By presenting our justification for each change and the outcome of those changes, we aim to demonstrate the varying impact of pilot iteration. Based on our findings, health equity-focused interventions are strengthened by repiloting. Repiloting provides stronger evidence and more support for scaling and health equity interventions, which may increase translation and sustainability. Future studies and investigators may consider objectively measuring the impact of changes made to their nurse-led interventions as they progress in their scaling.

## Data Availability

The datasets presented in this article are not readily available because maintaining participant confidentiality limits public access. Requests to access the datasets should be directed to Janiece L. Taylor, jwalke90@jhu.edu.
